# Social Vulnerability, Determinants for Equitable Health, and Cardiovascular Disease: A Bridge Still Too Far

**DOI:** 10.1016/j.jacadv.2024.100855

**Published:** 2024-03-06

**Authors:** Jamal S. Rana, Isaac Acquah

**Affiliations:** a Department of Cardiology, The Permanente Medical Group, Oakland, California, USA; b Division of Research, Kaiser Permanente Northern California, Oakland, California, USA; c Department of Medicine, MedStar Union Memorial Hospital, Baltimore, Maryland, USA

**Keywords:** cardiovascular disease, Social Determinants of Health, social vulnerability

**Figure:**
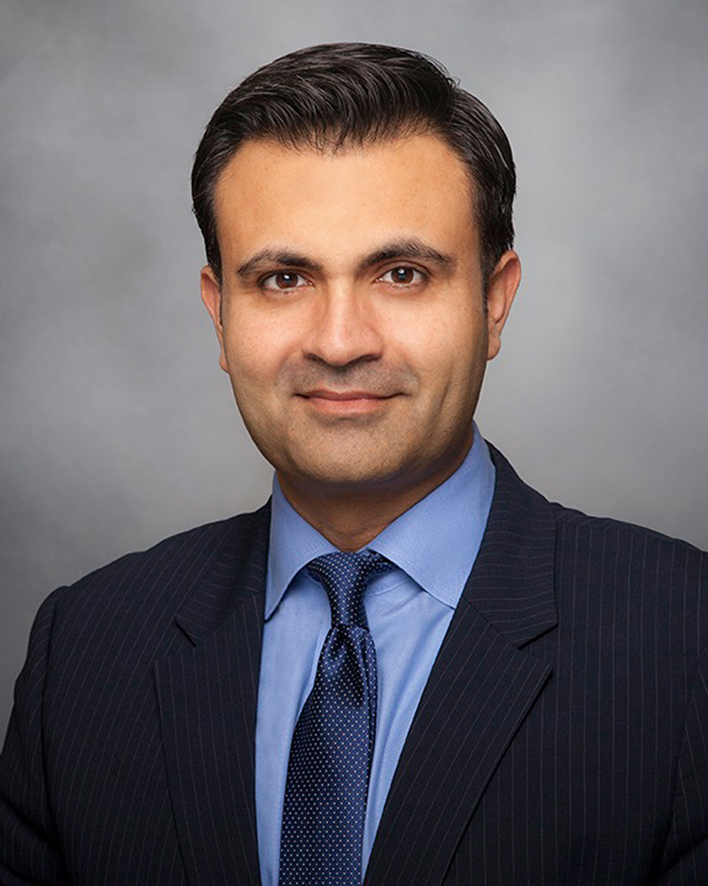

“Of all the forms of inequality, injustice in health care is the most shocking and inhumane.”

—Dr Martin Luther King, Jr

In the field of cardiovascular disease (CVD), much important work has been accomplished to address the challenge of ‘vulnerable plaque’. At the same time, we continue to live in a time where an individual’s zip code can dictate health outcomes.^1^ This calls for a foundational paradigm shift to also focus, identify, and care for ‘vulnerable patients’. The increasing attention on role of social determinants of health (SDOH) in CVD reflects a growing understanding that factors beyond medical care significantly influence CVD outcomes and create health disparities. SDOH are not isolated factors but interact and influence CVD risk at individual and community levels through a complex network of interconnected pathways. As a result, in our quest to understand and address cardiovascular vulnerability, it is crucial to focus on community-related social factors.

In this regard, researchers have developed various tools to assess SDOH at individual and community levels, including the area deprivation index and social vulnerability index (SVI). The tools provide a structured approach to quantifying these social vulnerabilities within specific communities, helping us better comprehend the challenges faced by individuals in those areas. The SVI, developed by the Centers for Disease Control and Prevention, quantifies the social needs of vulnerable communities at the county level, facilitating the identification of demographic groups and geographies susceptible to environmental and public health hazards. The SVI utilizes 16 U.S. census variables across 4 key SDOH domains: 1) socioeconomic status; 2) household composition and disability; 3) minority status and language; and 4) housing type and transportation. With higher scores representing the most vulnerable, the SVI helps identify communities requiring support before, during, or after disasters.

Leveraging insights from tools like the SVI, which serves as a practical guide for implementing meaningful interventions to empower us to take concrete steps in improving cardiovascular health within communities, is gaining significant interest among a wide range of stakeholders. Ibrahim et al^2^ deserve applause for delving deeper into the relationship between SVI and the CVD care continuum, as published in this issue of *JACC: Advances*. Through a systematic search of 7 databases, 1,812 records were initially identified. After removing duplicates and applying inclusion/exclusion criteria, 12 studies were included in the scoping review. These studies explored various aspects of the relationship between the SVI and CVD. Several studies found a positive association between higher SVI and CVD risk factor prevalence. Other studies demonstrated a link between higher SVI and CVD mortality outcomes. This review comprehensively examines the association between the SVI and the CVD care continuum, revealing crucial research gaps for future investigations. While 10 of the 12 studies in this study were cross-sectional and may, therefore, raise concerns about reverse causality, the findings of these associations were consistent even in the cohort studies identified. Furthermore, other longitudinal studies of various SDOH-CVD associations have shown similar results.^3^

Their consistent finding of an association between the SVI and various aspects of CVD across all 12 studies reviewed underscores the persistent role of SDOH in perpetuating health inequities and disease burden among vulnerable populations. Notably, this research extends the SVI's original purpose of identifying communities needing support during disasters to encompass CVD as a critical public health concern. For instance, a sharp increase in deaths from heart disease and stroke was noted during the pandemic, with greater increases among members of racial and ethnic minority groups, including a concerningly high increase among non-Hispanic Black individuals.^4^ These results brought out how COVID-19 magnified the underlying structural racism and systemic inequities. The pandemic seemed to melt the tip of the iceberg to reveal how precarious our health care system is for many populations, and that is where tools such as SVI can be valuable for upstream targeted interventions.

The United States spends nearly 18% of gross domestic product on health care, yet Americans die younger and are less healthy than residents of other high-income countries.^5^ Having the lowest life expectancy among high-income countries and the highest rate of avoidable deaths calls for an urgent need to evaluate the current economics of health care. Recent emphasis on policy shifts toward value-based care to achieve better population-level outcomes. Integrated health care models such as Kaiser Permanente have shown promise, where heart disease death rates among adults aged 45 to 65 fell by 48.3% in 3.2 million members from 2000 to 2015, compared to a 23.6% decline nationwide.^6^ On the other hand, there is sobering data from the National Health and Nutrition Examination Survey that over 2 decades in the U.S. income-based disparities in hypertension, diabetes, and smoking have persisted even after adjustment for other SDOH.^7^ Importantly, organizations are increasingly devoting time and resources to SDOH. It is an integral part of strategic planning for the American College of Cardiology. Similarly, the Division of Cardiovascular Sciences of the National Heart, Lung and Blood Institute includes “social determinants of cardiovascular health and health inequities” as a strategic vision.^8^

While the promise of using community-based social risk scores for transformative health care interventions is exciting, the path forward is not without its challenges. Navigating the landscape of validation, adoption, and the translation of findings into *actionable* care processes requires a concerted effort from all stakeholders involved. First, the consistent association between SVI and CVD across all studies highlights the need to explore specific pathways and mechanisms behind these links. Future research should focus on identifying how SDOH influences CVD risk and outcomes through longitudinal studies that can establish causality. While the SVI is a valuable tool, it was not designed for health care and may lack the sensitivity to detect all vulnerable groups. For instance, factors like discrimination, social support, and marital status, all of which significantly impact CVD risk, are not included in the SVI. The development of health care and CVD-specific SDOH assessment tools is crucial for accurately identifying vulnerable populations and designing targeted interventions.

Second, the concept of address-based social risk scores harmonizing various social determinants across diverse populations, each characterized by unique cultural, geographic, and socio-economic nuances will require robust validation studies comparing them at individual levels. Third, the journey from research to real-world implementation is often hindered by challenges related to adoption. Policymakers, health care systems, and practitioners must be willing to embrace the paradigm shifts toward a more holistic understanding of health disparities. Incorporating aggregated social risks into existing clinical workflows requires significant changes to data collection, documentation, and decision-making processes. Overcoming resistance to change and fostering a culture of inclusivity are vital steps in ensuring that these approaches become integral to ‘health care’ and not just ‘disease care’.

In conclusion, as we face this societal moral imperative while cognizant of the challenges, we must approach this journey with optimism. By filling research gaps, developing refined assessment tools, and embracing a comprehensive approach to health care, studies like Ibrahim et al advance our understanding of the SDOH-CVD bridge. The zip code and address go beyond their numerical designation to symbolize localized disparities and opportunities. As we unravel the complexities of aggregating social risks, the community conditions emerge as a powerful instrument that empowers us to dissect and address health inequalities within specific local environments. By focusing on these geographical identifiers, we gain the insights necessary to dismantle systemic barriers, implement targeted interventions, and pave the way for a future where health outcomes are no longer determined solely by geographical coordinates. We are extremely hopeful that such knowledge will empower us to design effective interventions and ultimately reduce health disparities related to CVD. In doing so, we honor Dr King's call for justice in health care and take a step closer to achieving equitable health for all.

## Funding support and author disclosures

The authors have reported that they have no relationships relevant to the contents of this paper to disclose.
